# Supplementary material for: Different Effects of RNAi-Mediated Downregulation or Chemical Inhibition of NAMPT in an Isogenic IDH Mutant and Wild-Type Glioma Cell Model
Source: Int J Mol Sci. 2022 May 21;23(10):5787. doi: 10.3390/ijms23105787 (PMC9143996; doi:10.3390/ijms23105787)
Supplement: Supplementary file 1 [file ijms-23-05787-s001.zip › ijms-1703753-supplementary.pdf]

Supplementary Material

Different effects of RNAi-mediated downregulation or chemical inhibition of NAMPT in IDH mutant and wild-type glioma cells

Maximilian Clausing[1,2], Doreen William[1,3], Matthias Preussler[1,2], Julia Biedermann[1,2], Konrad Grützmann[3], Susan Richter[4], Frank Buchholz[5], Achim Temme[2,6], Evelin Schröck[1,3], Barbara Klink[1,3,7,*]

[1] Institute for Clinical Genetics, University Hospital Carl Gustav Carus at the Technische Universität Dresden, 01307 Dresden, Germany, ERN-GENTURIS, Hereditary Cancer Syndrome Center Dresden, Germany

[2] National Center for Tumor Diseases partner site Dresden (NCT/UCC), Germany, German Cancer Consortium (DKTK), Dresden, Germany; German Cancer Research Center (DKFZ), Heidelberg, Germany

[3] Core Unit for Molecular Tumor Diagnostics (CMTD), National Center for Tumor Diseases (NCT/UCC), Dresden, Germany, German Cancer Consortium (DKTK), Dresden, Germany; German Cancer Research Center (DKFZ), Heidelberg, Germany

[4] Institute for Clinical Chemistry and Laboratory Medicine, University Hospital Carl Gustav Carus, Technische Universität Dresden, 01307 Dresden, Germany

[5] Medical Systems Biology, Faculty of Medicine and University Hospital Carl Gustav Carus, Technische Universität Dresden, 01307 Dresden, Germany

[6] Department of Neurosurgery, Section Experimental Neurosurgery/Tumor Immunology, University Hospital Carl Gustav Carus, Technische Universität Dresden, 01307 Dresden, Germany

[7] National Center of Genetics (NCG), Laboratoire National de Santé (LNS), 1, Rue Louis Rech, L-3555 Dudelange, Luxembourg

[*] Correspondence: barbara.klink@lns.etat.lu

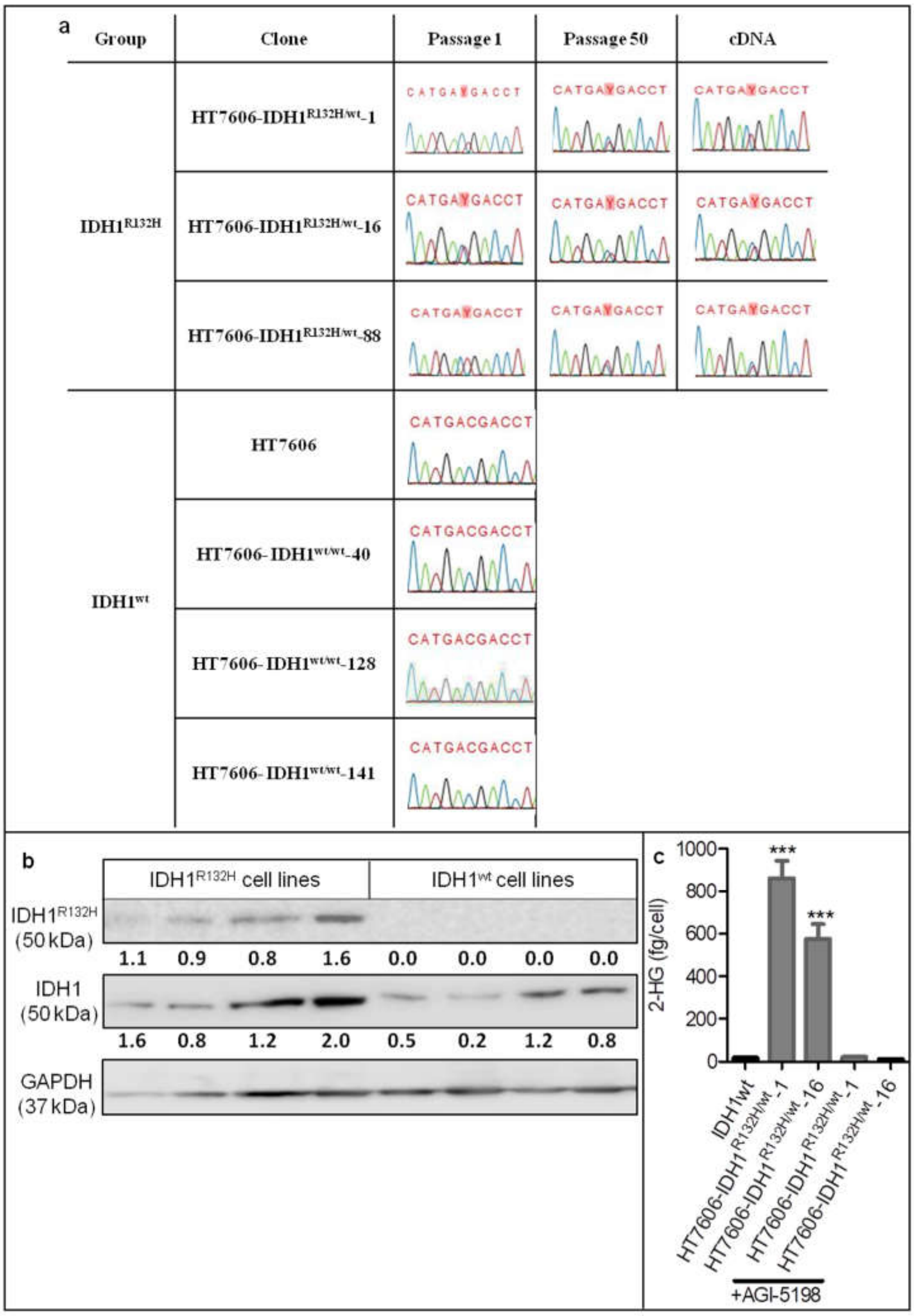

Supplementary Figure S1. (**a**) genomic status of edited IDH1 c.395G>A clones. All edited clones were sequenced for the IDH1 c.395G>A Mutation. The heterozygous mutation remained over a period of 50 passages and was present in a heterozygous state on cDNA level confirming the transcription of the mutated allele. HT7606-IDH1$^{R132H/wt}$-16 was created using the Cas9-NLS-tagRFP. The remaining cell lines were created using the Cas9-plasmid pX458. (**b**) Western Blot analyses of IDH1 and IDH1^{R132H} in IDH1^{wt} cell lines (HT7606, HT7606-IDH1$^{wt/wt}$-128) and IDH1^{R132H} cell lines (HT7606-IDH1$^{R132H/wt}$-88, HT7606-IDH1$^{R132H/wt}$-1, HT7606-IDH1$^{R132H/wt}$-16). GAPDH was used as protein loading control. Data present the ratio of NAMPT to GAPDH. (**c**) 2-HG levels were measured using liquid chromatography-tandem mass spectrometry in untreated IDH1^{wt} and IDH1^{R132H} cells and after treatment with the selective mutant IDH1 inhibitor AGI-5198 for 48 hours (n_b for IDH1^{wt}=3; n_t=2; *** $p \leq 0.001$).

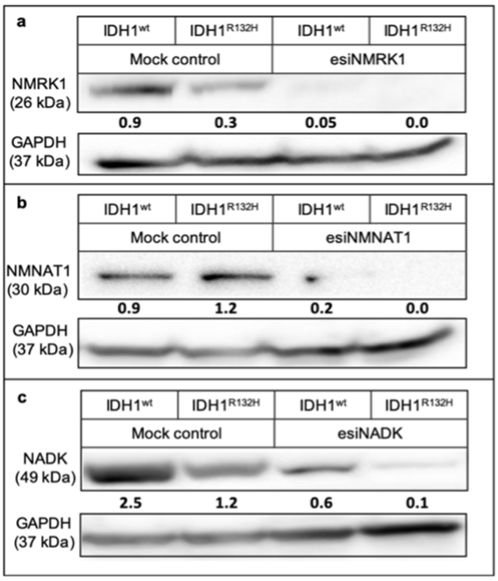

Supplementary Figure S2. Western blot analyses of NMRK1 (**a**), NMNAT1 (**b**) and NADK (**c**) in IDH1^{wt} (HT7606-IDH1$^{wt/wt}$-40) and IDH1^{R132H} cells (HT7606-IDH1$^{R132H/wt}$-1) after treatment with mock control, esiNMRK1 (**a**), esiNMNAT (**b**) or esiNADK (**c**) for 72 hours. GAPDH was used as protein loading control. Data present the ratio of NAMPT to GAPDH. (n_t=3).

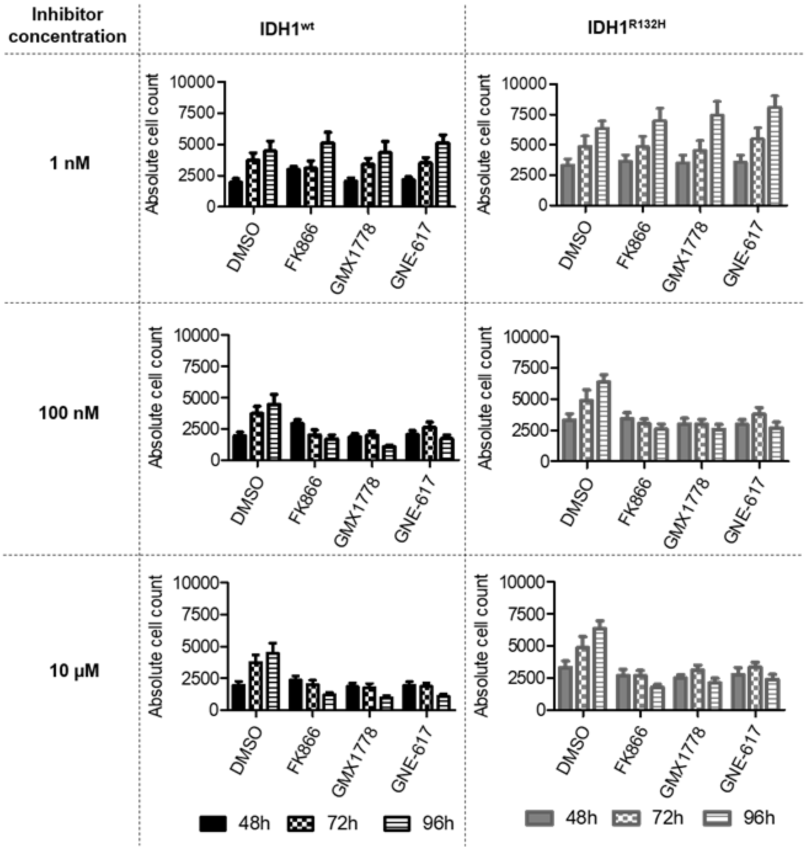

Supplementary Figure S3. Low concentrations of NAMPT inhibitors are not sufficient to induce cytotoxicity in IDH1^{wt} or IDH1^{R132H} cells. Cell count was determined by live and dead staining with Hoechst and Propidium Iodide with the Operetta Imaging system after treatment with 1 nM, 100 nM or 10 µM of FK866, GMX1778 and GNE-617 for 48, 72 or 96 hours.(n_b=3 per group; n_t=3).

Supplementary Table S1. Overview of oligonucleotides.

Name	Sequence (5′ to 3′)	length (nt)	Purpose
IDH1_395A_F	GGGTAAAACCTATCATCATAGGTAA	164	Allele-specific PCR after CRISPR/Cas
IDH1_395G_F	GGGTAAAACCTATCATCATAGGTAG		
IDH1_395_R	TCATACCTTGCTTAATGGGT		
IDH1_ex4_2_R	CCAAGTCACCAAGGATGCTG	467	IDH1 Sanger-Sequencing
IDH1_in4_2_F	TGTTGAGATGGACGCCTATTTG		
cIDH1_ex4_F1	GGCCAACCCTTAGACAGAG	450	IDH1 cDNA Sanger-Sequencing
cIDH1_ex6_R	CACCAAGGATGCTGCAGAAG		
ATP1A1_sgRNA5_OT8_F	ATGCAGGTGAAGAGCCAGAG	246	CRISPR/Cas gRNA off-targets screening
ATP1A1_sgRNA5_OT8_R	GGCAAAGGGTTTACAAATCACC		
C21orf15_sgRNA5_2_F	GTCCATGGTGTTGACTGCTC	193	
C21orf15_sgRNA5_2_R	TCCCCAGAAACATCCACAGA		
CHI3L2_sgRNA5_OT5_F	TGCTTCTTTCCAGGTTCCAC	216	
CHI3L2_sgRNA5_OT5_R	CTGAGTACACCGTGGCATCT		
FAM95C_sgRNA5_OT7_F	AACCTCTGACTCTGCCTTGG	296	
FAM95C_OT7_2_R	TAAGTGGTTGCCGTTTGTGG		
FAT2_sgRNA5_OT4_2_F	GGCCCCACAGAGAAAACAAG	219	
FAT2_sgRNA5_OT4_R	TTGGGCACTTCACCATTCAC		
MME_sgRNA5_OT10_F	TGCTTTAGGTGCTTCTTGGA	280	
MME_sgRNA5_OT10_R	CCAGGTATTAGGGACAGAGCA		
NEB_sgRNA5_OT14_F	TGCAGGAATGAGGAAGAGCA	268	
NEB_sgRNA5_OT14_R	AGATGTCAGCTACCACACCT		
NFE2L1_sgRNA5_OT11_F	GACACTCTGACCCAAGACGA	217	
NFE2L1_sgRNA5_2_R	CTGGCAGAACACAAGGGATG		
ORC2_sgRNA5_OT3_F	TCCCAGAGCAGTGAACAAGT	262	
ORC2_sgRNA5_OT3_R	AGCAGCTCTACAGATCCACT		
PGK1_sgRNA5_OT9_2_F	GCCTTACAGTTTTGGTGCCA	241	
PGK1_sgRNA5_OT9_R	TTCTACCCACCTCTCACTGC		
PHGDH_sgRNA5_OT2_F	AGCTGAGAAACTCCAGGTGG	253	
PHGDH_sgRNA5_OT2_R	TGCTGTGTGTGTTGACCATG		
PRR14L_sgRNA5_OT1_F	GCAACAGAGCAGTACCTTGC	246	
PRR14L_sgRNA5_OT1_R	TGCAGATTGAAGAGGCAGGA		
TMEM184A_sgRNA5_ OT12_F	CGCAGATTTGAGCAACTTGAC	308	
TMEM184A_sgRNA5_ OT12_R	TCTTGCTTCCTGGGCTGAG		
WIPF2_sgRNA5_OT13_F	GCTGCTCTCGAACTCCTGA	306	
WIPF2_sgRNA5_OT13_R	CTCCTTGGAAGAGACCTCCC		
cNAMPT_ex1_F	GGCAGAAGCCGAGTTCAA	82	RT-qPCR measurement
cNAMPT_ex2_R	GCTTGTGTTGGGTGGATATTG		
ARF1_qPCR_F	GACCACGATCCTCTACAAGC	111	
ARF1_qPCR_R	TCCCACACAGTGAAGCTGATG		
GAPDH_2_F	ATGTTCGTCATGGGTGTGAA	89	
GAPDH_2_R	GGTGCTAAGCAGTTGGTGGT		

Supplementary Table S2. Possible off-target regions of the specific gRNA used in the CRISPR/Cas experiments that were predicted with crispr.mit.edu. All off-target sequences located in genes are listed and were negatively screened for in all clones using Sanger Sequencing.

Gene name	Sequence	Locus
CHI3L2	ATGAATCAAGTAATTCATGTGAG	chr1:+111777685
ATP1A1	GGGGATGAAGTAAGTAATGAAGG	chr1:+116943872
PHGDH	GGTTATGAAGTAAGTCATGGAGG	chr1:+120263934
NEB	GGGCATCAAGTAACTGATCTGAG	chr2:+152406288
ORC2	AAGGAACAAGTAAGTCATGAGAG	chr2:+201778278
MME	GTGAATCATGTAATTCATGTAAG	chr3:+154834756
FAT2	CGGGAACAGGTGAGTCATGTGGG	chr5:-150907538
TMEM184A	GGGGATCCAATAAGACAGGTGGG	chr7:+1584857
FAM95C	AGGCATCAAGTAAGGCCTGTGGG	chr9:-38542926
WIPF2	GGTGACCAAGTAAATCATGAGGG	chr17:-38418704
NFE2L1	GTGGAGAAAGTAAGTCACGTGGG	chr17:+46128171
C21orf15	AGGCATCAAGTAAGTCTTATGGG	chr21:-15220392
PRR14L	GAGGATCAAATAAGTCCTGTAAG	chr22:-32112066
PGK1	GAGGATAAAGTCAGCCATGTGAG	chrX:+77380863
